# Editorial: Signaling Pathways in Developing and Pathological Tissues and Organs of the Craniofacial Complex

**DOI:** 10.3389/fphys.2018.01015

**Published:** 2018-07-26

**Authors:** Thimios A. Mitsiadis, Claudio Cantù, Lucia Jimenez-Rojo

**Affiliations:** ^1^Orofacial Development and Regeneration, Centre for Dental Medicine, Institute of Oral Biology, University of Zurich, Zurich, Switzerland; ^2^Department of Clinical and Experimental Medicine, Faculty of Health Sciences, Wallenberg Centre for Molecular Medicine, Linköping University, Linköping, Sweden

**Keywords:** signaling pathways, tooth, enamel, palate, calvaria, craniofacial, stem cells, tissue regeneration

The field of craniofacial biology has greatly benefited from the progress made the last decades in developmental biology, molecular biology, stem cell biology, and genomics. A detailed description of the action of signaling pathways that are involved in developmental processes has significantly increased our comprehension of the organization and function of molecular networks that are involved in the formation of the craniofacial complex (Kouskoura et al., 2011). Craniofacial development requires the patterning, outgrowth, fusion, and molding of various tissues with a heterogeneous developmental origin. The important role of signaling molecules driving epithelial-mesenchymal interactions in craniofacial development has been revealed by sophisticated genetic manipulations in mice (Cobourne and Mitsiadis, 2006).

The importance of the various signaling pathways in embryonic development was revealed more than two decades ago. Since then, the concept of how signaling molecules bind to their receptors and are transported from the membrane to the cytoplasm and/or nucleus has been corroborated with new exciting findings. The multifunctional nature of the signaling molecules provides craniofacial tissues and organs with versatile means of driving their development and controlling cell behavior. These molecules have numerous effects on cell proliferation, migration, differentiation, tissue morphogenesis, homeostasis and regeneration, and their deregulation and malfunction lead to severe craniofacial pathologies. The effects of the signaling molecules can be different, even opposite, depending on the cell type on which they act, and the environmental conditions. Elucidating the functionality of the various signaling pathways raised the specter of complicated signal transduction processes (Kouskoura et al., 2011). The biochemical crosstalk between molecules previously considered as being dedicated only to specific signaling pathways revealed new principles of signal-driven transcriptional action. Target genes of the various signaling molecules may trigger stem cell differentiation, cell cycle arrest, apoptosis, and even immune responses. It is important to elucidate how cells transit from one state to another (e.g., from stem cells to differentiated cells) under the governance of precise signaling molecules (Mitsiadis et al., 2007).

Recent advances have showed the importance of signaling molecules in craniofacial physiology and disease and moved the field closer to a more comprehensive understanding of the context-dependent nature of their action. Mutations in regulators and selected components of the signaling pathways were a first indication of their medical relevance (Kouskoura et al., 2011). Research on the ligand-receptor and adaptor protein-transcription factors interactions will generate important knowledge aimed at the development of new pharmaceutical products that mimic and block these interactions (Yong-Ming et al., 2017; Zimmerli et al., 2017; Aung et al., 2018). Finally, understanding how and when the various signaling pathways may activate cell proliferation, migration and apoptosis could provide impetus to the development of new therapeutic approaches after injury or cancer within the craniofacial complex (Pagella et al., 2017).

In this research topic, prominent researchers within the craniofacial field have contributed with important discoveries and generated exciting results concerning the role of signaling pathways in the pathophysiology of the various craniofacial tissues. Numerous original articles have evidenced the big variety of signals that are necessary for controlling developmental and pathological events of the craniofacial organs.

The influence of systemic glucocorticoid administration in the composition of bone structures of the jaw of mini pigs is reported (Schulz et al.). The ion channel protein TRPM7 mediates the mineralization process of craniofacial hard tissues (Nakano et al.). The influence of photobiomodulation on the capacity of bone marrow cells to form alveolar and craniofacial bones is also discussed (Amaroli et al.). Calvaria formation is a complex process that necessitates the coordinated action of osteogenic stem cell populations (Doro et al.), controlled by specific signaling molecules such as Indian Hedgehog and the transcription factor Gli3 (Veistinen et al.). Palatogenesis is an equally complex process orchestrated by cytoskeleton and extracellular matrix rearrangement (Chiquet et al.) and expression of specific signaling molecules (Iyyanar and Nazarali; Xavier et al.).

The identification of stem cell compartments in conjunction with the discovery of the complex ensemble of signals that creates their microenvironment is essential for a successful regenerative approach. Therefore, the identification and characterization of stem cell niches within dental pulp and the analysis of Notch signals during tooth repair is of prime importance (Mitsiadis et al.).

Loss of the alveolar bone that surrounds teeth could be an actor of periodontal pathology, and this process could be controlled via the *Stemodia maritime* L. antioxidant extracts (Teixeira et al.). Similarly, Wnt signaling (Mitsiadis et al.), VEGF and Hypoxia Inducible Factor (Oishi et al.) and Syndecan-1 (Filatova et al.) might affect alveolar bone remodeling and periodontal ligament homeostasis.

Defined molecular mechanisms such as GLI-mediated transcription (Chang et al.), Fibroblast Growth Factor 10 (Teshima et al.), MORN5 (Cela et al.) and the microRNA Mir23b and Mir133b (Ding et al.) are involved in the physiopathology of a variety of tissues and organs of the craniofacial complex. The Wnt pathway controls the growth of dental lamina (Putnová et al.) from where the future teeth will develop. Retinoic acid (Morkmued et al.), steroids (Houari et al.), and Na:K:2Cl Cotransporter-1 (Jalali et al.) control the formation of enamel. Generation of different dental epithelial spheres that contain stem cells might enhance our understanding of enamel formation (Natsiou et al.). Dentin sialophosphoprotein (DSPP) is also very important for proper dentin formation and mutations in the *DSPP* gene lead to dentinogenesis imperfecta (Bloch-Zupan et al.). The role of Nerve Growth Factor pathway is similarly important during human tooth development and repair (Mitsiadis and Pagella). Finally, the Hippo-YAP1/TAZ cascade is important for pituitary stem cell development (Lodge et al.).

The increased knowledge on the development, pathology and regeneration of tissues and organs of the craniofacial complex will most certainly orchestrate a significant shift toward novel diagnostic and therapeutic approaches. Although many questions concerning the mechanisms involved in craniofacial tissues development and regeneration have not yet been resolved, modern imaging tools, mathematics, bioinformatics, and genomics will help to elaborate new concepts and models that will change drastically this field. Further progress in treatments concerning craniofacial tissues and organs depends upon active and vigorous research programs.

## Author contributions

All authors listed have made a substantial, direct and intellectual contribution to the work, and approved it for publication.

### Conflict of interest statement

The authors declare that the research was conducted in the absence of any commercial or financial relationships that could be construed as a potential conflict of interest.
